# Socioeconomic inequalities in stillbirth rates in Europe: measuring the gap using routine data from the Euro-Peristat Project

**DOI:** 10.1186/s12884-016-0804-4

**Published:** 2016-01-19

**Authors:** Jennifer Zeitlin, Laust Mortensen, Caroline Prunet, Alison Macfarlane, Ashna D. Hindori-Mohangoo, Mika Gissler, Katarzyna Szamotulska, Karin van der Pal, Francisco Bolumar, Anne-Marie Nybo Andersen, Helga Sól Ólafsdóttir, Wei-Hong Zhang, Béatrice Blondel, Sophie Alexander, Euro-Peristat Scientific Committee

**Affiliations:** Inserm UMR 1153, Obstetrical, Perinatal and Pediatric Epidemiology Research Team (Epopé), Center for Epidemiology and Statistics Sorbonne Paris Cité, DHU Risks in pregnancy, Paris Descartes University, 53 avenue de l’Observatoire, 75014 Paris, France; Department of Public Health, University of Copenhagen, Copenhagen, Denmark; Centre for Maternal and Child Health Research, City University London, London, England; Department of Child Health, TNO, Netherlands Organisation for Applied Scientific Research, Leiden, The Netherlands; THL National Institute for Health and Welfare, Helsinki, Finland; Department of Epidemiology, National Research Institute of Mother and Child, Kasprzaka 17 a, 01-211 Warsaw, Poland; Department of Public Health Sciences, University of Alcalá, Madrid, Spain; Department of Obstetrics and Gynaecology, Landspitali University Hospital, Landspitali v/ Hringbraut, Reykjavík, Iceland; Perinatal Epidemiology and Reproductive Health Unit, Epidemiology, Biostatistics and Clinical Research Centre, School of Public Health, Université Libre de Bruxelles, Brussels, Belgium

**Keywords:** Stillbirth, Socioeconomic factors, Europe, Health inequalities, Pregnancy, Mortality

## Abstract

**Background:**

Previous studies have shown that socioeconomic position is inversely associated with stillbirth risk, but the impact on national rates in Europe is not known. We aimed to assess the magnitude of social inequalities in stillbirth rates in European countries using indicators generated from routine monitoring systems.

**Methods:**

Aggregated data on the number of stillbirths and live births for the year 2010 were collected for three socioeconomic indicators (mothers’ educational level, mothers’ and fathers’ occupational group) from 29 European countries participating in the Euro-Peristat project. Educational categories were coded using the International Standard Classification of Education (ISCED) and analysed as: primary/lower secondary, upper secondary and postsecondary. Parents’ occupations were grouped using International Standard Classification of Occupations (ISCO-08) major groups and then coded into 4 categories: No occupation or student, Skilled/ unskilled workers, Technicians/clerical/service occupations and Managers/professionals. We calculated risk ratios (RR) for stillbirth by each occupational group as well as the percentage population attributable risks using the most advantaged category as the reference (post-secondary education and professional/managerial occupations).

**Results:**

Data on stillbirth rates by mothers’ education were available in 19 countries and by mothers’ and fathers’ occupations in 13 countries. In countries with these data, the median RR of stillbirth for women with primary and lower secondary education compared to women with postsecondary education was 1.9 (interquartile range (IQR): 1.5 to 2.4) and 1.4 (IQR: 1.2 to 1.6), respectively. For mothers’ occupations, the median RR comparing outcomes among manual workers with managers and professionals was 1.6 (IQR: 1.0–2.1) whereas for fathers’ occupations, the median RR was 1.4 (IQR: 1.2–1.8). When applied to the entire set of countries with data about mothers’ education, 1606 out of 6337 stillbirths (25 %) would not have occurred if stillbirth rates for all women were the same as for women with post-secondary education in their country.

**Conclusions:**

Data on stillbirths and socioeconomic status from routine systems showed widespread and consistent socioeconomic inequalities in stillbirth rates in Europe. Further research is needed to better understand differences between countries in the magnitude of the socioeconomic gradient.

**Electronic supplementary material:**

The online version of this article (doi:10.1186/s12884-016-0804-4) contains supplementary material, which is available to authorized users.

## Background

Socioeconomic disadvantage, as measured by low levels of mothers’ or fathers’ education, occupational status or income, is associated with raised risks of stillbirth even in countries with universal insurance coverage and generous welfare provisions [1–6]. The hypothesised mechanisms are multiple and interrelated and include smoking, poor diet and other unhealthy behaviours, higher stress, less social support and depression, teenage motherhood, unplanned pregnancies, a higher prevalence of chronic health conditions, such as hypertension, diabetes, or obesity as well as poor access to antenatal care and receipt of suboptimal care [1, 7–9].

Many questions remain surrounding the causes of stillbirth and effective approaches to prevention. A high proportion of stillbirths, up to 30 %, remain unexplained even with complete clinical and autopsy records [10, 11]. Further, most stillbirths in high income countries occur before the onset of labour [12], providing fewer obvious possibilities for medical intervention than, for instance, cases of intra-partum, neonatal or infant death. Investigating the associations between socioeconomic position and stillbirth risk is an important component of efforts to advance knowledge about the aetiology and prevention of stillbirth.

A focus on social inequalities also emphasises the distal determinants of stillbirth risk which are accumulated over the life course and relate to parental health status, behaviours and knowledge preceding the pregnancy. Stillbirths may also perpetuate social differences in health by creating disease, in particular, maternal depression [13], and have been associated with long term risks for the mother [14]. Improving pregnancy outcomes by reducing stillbirths is therefore a component of broader national strategies to interrupt the transgenerational cycle of ill-health.

Comparing outcomes between social groups illustrates the poorer health associated with socioeconomic disadvantage and the outcomes that could be achieved in the absence of these inequalities. Having reliable indicators for measuring social inequalities in stillbirth enables target-setting for health policies, performance benchmarking and monitoring of trends over time. In this study, we sought to assess the magnitude of social inequalities in stillbirth rates in European countries using indicators generated from routine monitoring systems.

## Methods

### Data sources

Data come from the Euro-Peristat project, a collaboration between 26 member states of the European Union and Norway, Iceland and Switzerland, to assess perinatal health in Europe using a common set of 10 core and 20 recommended perinatal health indicators [15–17]. The indicators were developed following several Delphi consensus processes with multidisciplinary and geographically diverse panels of experts [18]. The aim was to select a succinct set of comparable indicators of mothers’ and newborn babies’ health and care which can be monitored routinely.

Data for the Euro-Peristat indicators were compiled using nationally aggregated population-based data from routine data collection systems for the year 2010, and have been described previously [15, 17, 19]. One scientific committee (SC) member per country was responsible for the oversight of data collection in their country, in collaboration with other data providers. When national data were not available, population-based regional data could be provided. In Belgium, data were provided separately for Brussels, Flanders and Wallonia and in the UK for England and Wales, Northern Ireland and Scotland. Slovakia provided core, but no recommended indicators. As these include the socioeconomic indicators, it could not be included in this study. In most countries, the data came from medical birth registers, civil registration and child health systems. Data in France and Cyprus came from routine surveys used to monitor perinatal health. In Cyprus the survey took place in 2007. In France, this source was used because of changes to definitions of stillbirth in 2007 meant that comparable indicators of stillbirths were not available from vital statistics in 2010 [20].

This study is based on aggregated routinely collected data, so ethics approval was not required.

### Socioeconomic factors

Mothers’ level of education was selected as the Euro-Peristat marker of socioeconomic position, based on the Delphi process, which ranked indicators on experts’ opinions about relevance as well as feasibility [18]. As an indicator for international comparisons, educational level has the advantage that UNESCO has established an international classification, the International Standard Classification of Education (ISCED), which has also been adopted by the EU Directorate General for Education and Culture [21]. However, because some European countries do not routinely collect data on education, updates to the Euro-Peristat indicator set in 2012 added SES indicators based on mothers’ and fathers’ occupational group.

The mothers’ level of education indicator is defined as the highest level of education of women delivering live or stillborn babies. Data were requested on most detailed educational groupings in national systems and then recoded using the International Standard Classification of Education (ISCED - UNESCO, 1997), as follows: (0) primary not complete or none (1) primary complete, (2) lower secondary (up to 3 or 4 years), (3) higher secondary (up to 6 or 7 years), (4) post-secondary non tertiary (6 months to 2 years), (5) first stage of tertiary education (bachelor), (6) second stage of tertiary education (master, doctorate or more), (9) unknown. These categories were then collapsed into three groups: none, primary and lower secondary (0–2), higher secondary (3) and post-secondary (4–6). After the national classifications were recoded into the ISCED categories, they were sent to the SC members for validation.

Mother’s and father’s occupational class for parents of live or stillborn babies was defined using the International Standard Classification of Occupations (ISCO-08) major groups: (1) managers (2) professionals (3) technicians and associate professionals (4) clerical support workers (5) service and sales workers (6) skilled agricultural, forestry and fishery workers (7) craft and related trades workers (8) plant and machine operators, and assemblers (9) elementary occupations (0) armed forces occupations (99) no profession (88) student. These occupational groups were then amalgamated into four categories: no occupation or student, skilled/unskilled workers, technicians/clerical/service occupations and managers/professionals. These were ordered from lowest to highest social status to be consistent with the coding of educational level. When countries could not provide these categories, they included those that they used. Ireland’s classification is not based on the ISCO-08, but was recoded into the four groups for our analysis. Occupations for England and Wales had been grouped into the eight class version of the National Statistics Socio-economics Classification (NSSec) [22] and subdivided into births registered jointly by both parents and those grouped by the mother alone. Students were included with”occupations inadequately described; occupations not classifiable for other reasons; never worked and long-term unemployed.” This category was classified as unknown for the analysis. In Luxembourg, the classification is based on parent’s employment (employed, student or unemployed) and these data were not used. In Slovenia, not all maternity units used the ISCO classification and these partial data were not used.

### Stillbirths

Aggregated data were collected on the numbers of stillbirths and live births grouped by the three socioeconomic indicators: mother’s level of education and mother’s and father’s occupational group. Euro-Peristat defines the stillbirth rate as the number of stillbirths at or after 22 completed weeks of gestation in a given year per 1000 live and stillbirths in the same year. If gestational age was not available, inclusion was determined by a birthweight of 500 grams or more. Not all countries could provide stillbirth data using this definition, as this depends on national registration criteria. In these cases, countries provided numbers of stillbirths in accordance with national definitions. Lower thresholds were 500 g in Germany, Austria, Poland, Slovenia, 24 weeks in Greece, Portugal and the United Kingdom (although Portugal and Scotland have voluntary notification at 22/23 weeks), 180 days in Spain and 500 grams or 24 weeks in Ireland. Another difference between countries is the way terminations of pregnancy (TOP) are recorded (either not at all, in a separate register or as stillbirths). In Slovenia and France, TOP were removed for this analysis (see Additional file 1: Table S1 for more details about inclusions).

### Analysis

We described the availability of data on mother’s level of education and parents’ occupational groups for monitoring social differences in stillbirth and compared the distribution of these variables between the countries. Missing data were reported as a separate category and excluded from the analyses. We then calculated stillbirth rates for each educational and occupational category and corresponding risk ratios (RR) using the highest category as the reference (post-secondary education and professional/managerial occupations). To assess the potential margin for improvement, we calculated the percentage population attributable risks (%PAR) and their 95 % confidence intervals in each country using the best educated or highest occupational category as the reference. These were then applied to the number of stillbirths in our study to derive a predicted number of stillbirths if rates for all women were the same as the rates for the highest SES categories in their country. Results are also summarised as medians and intra-quartile ranges to provide general estimates and the spread across the counties. To assess the potential impact of missing data on our estimates, we calculated the association of the RR of the lowest social category to the reference group for each indicator with the proportion of missing data using Spearman correlation coefficients. Data were analysed with Stata SE13.0 and R 3.1.3 software.

## Results

### Data availability and comparability

As shown in Table 1, 23 out of 29 (79 %) European countries provided stillbirth rates by at least one of the three SES indicators. Data on stillbirth rates by mother’s level of education were available in 19 countries and by mother’s and father’s occupation in 13 countries. Nine countries provided stillbirth data grouped by both indicators.

**Table 1:** Countries providing data and classifications used

	Mother’s level of education			Mother’s occupation		Father’s occupation	
Country	Availability	Missing data (%)	Number of class^a^	Availability	Missing data (%)	Availability	Missing data (%)
BE: Brussels	Yes	8	8	Yes	1	Yes	8
BE: Flanders	Yes	9	13	Yes	4	Yes	4
BE: Wallonia	Yes	23	8	Yes	5	Yes	18
Czech Republic	Yes	7	5	--	--	--	--
Denmark	Yes	5	10	--	--	--	--
Germany	--	--	--	Yes	22	--	--
Estonia	Yes	0	7	Yes	25	Yes	21
Ireland	--	--	--	Yes	5	Yes	26
Greece	--	--	--	--	--	--	--
Spain	Yes	5	11	Yes	5	Yes	8
France	Yes	4	7	Yes	3	Yes	7
Italy	Yes	3	6	--	--	--	--
Cyprus	Yes	1	9	--	--	--	--
Latvia	Yes	0	7	--	--	--	--
Lithuania	Yes	0	6	Yes	0	--	--
Luxembourg	Yes	4	8	--	--	--	--
Hungary	Yes	1	6	--	--	--	--
Malta	Yes	26	4	--	--	--	--
Netherlands	--	--	--	--	--	--	--
Austria	Yes	9	5	--	--	--	--
Poland	Yes	0	8	--	--	--	--
Portugal	Yes	2	9	Yes	13	Yes	10
Romania	--	--	--	Yes	0	--	--
Slovenia	Yes	15	12	--	--	--	--
Slovakia	--	--	--	--	--	--	--
Finland	Yes	14	7	Yes	27	Yes	13
Sweden	--	--	--	--	--	--	--
United Kingdom	--	--	--	--	--	--	--
UK: England and Wales	--	--	--	--	--	Yes	12^b^
UK: Scotland	--	--	--	--	--	--	--
UK: Northern Ireland	--	--	--	--	--	--	--
Iceland	--	--	--	--	--	--	--
Norway	Yes	0	7	--	--	--	--
Switzerland	--	--	--	--	--	--	--

^a^Unknown included

^b^'Inadequately described or not in employment, including students'

All national educational classifications could be mapped into ISCED-97 codes, although countries provided varying levels of detail (from 4 to 13 categories) some did not record primary schooling only (Austria, Finland). Missing data ranged from 0 to 25 %, but was under 10 % in the majority of countries. In general, missing data were more common for stillbirths than for live births. As shown in Table 2, there were marked variations in the distribution of mothers’ level of education: primary and lower secondary (from 0.3 to 40 % of mothers), higher secondary (from 20 to 60 % of mothers) and post-secondary (from 20 to 60 % of mothers).

**Table 2:** Percentage distribution of mother’s levels of education and stillbirth rates by educational level

		Distribution of births				Stillbirth rates			
Country	Total Births	Primary and lower secondary	Higher secondary	Post-secondary	Total Stillbirths (SB)	Overall SB rate	SB rate Primay and lower secondary	SB rate Higher secondary	SB rate Post-secondary
	N (all stated)	%	%	%	N	*p* 1000	*p* 1000	*p* 1000	*p* 1000
Austria	72069	16.9	46.1	37.0	227	3.1	4.9	3.3	2.1
BE: Brussels	22965	22.4	37.1	40.5	44	1.9	1.9	2.6	1.3
BE: Flanders	62438	12.2	39.3	48.5	179	2.9	3.8	3.6	2.0
BE: Wallonia	29546	17.8	39.9	42.2	75	2.5	4.7	2.4	1.8
Cyprus	8302	9.5	29.8	60.6	25	3.0	2.5	4.8	2.2
Czech Republic	108843	10.9	42.9	46.2	191	1.8	3.8	1.6	1.5
Denmark	58841	16.6	36.7	46.6	220	3.7	5.7	3.9	2.9
Estonia	15613	14.2	46.2	39.6	61	3.9	7.2	4.0	2.6
Finland	51775	--	46.2	53.8	152	2.9	--	3.3	2.6
France	14060	28.3	19.9	51.8	61	4.3	6.0	5.0	3.2
Hungary	89979	20.0	46.3	33.7	349	3.9	8.2	3.5	1.8
Italy	529166	33.0	44.2	22.7	1388	2.6	3.2	2.4	2.2
Latvia	19246	16.3	39.6	44.1	107	5.6	7.0	6.0	4.6
Lithuania	30472	12.9	29.1	58.0	136	4.5	6.1	5.2	3.7
Luxembourg	6082	21.8	30.9	47.4	29	4.8	5.3	4.8	4.5
Malta	2987	0.3	63.9	35.8	11	3.7	0.0	4.2	2.8
Norway	53705	18.7	29.0	52.3	162	3.0	3.8	3.2	2.7
Poland	408878	8.8	49.7	41.6	1629	4.0	6.5	4.6	2.8
Portugal	98618	18.1	51.2	30.7	227	2.3	3.3	2.5	1.4
Slovenia	19074	17.3	46.0	36.7	74	3.9	4.5	3.4	4.1
Spain	464657	38.3	28.4	33.2	990	2.1	2.8	1.8	1.6

For fathers’ occupations, the skilled/unskilled worker category ranged from 22 to 47 %, but was between 35 and 45 % in most countries (Table 3); the intermediate occupational category varied between 20 and 43 % and the professionals and managers from 12 to 43 %. No occupation/student varied from 2 to 21 %, but in most countries was close to 5 %. It was unclear how father’s occupation was coded if the father was not named on the birth registration, as there was no separate category in most classifications. Only two countries provided a category “Sole registration by mother” (England and Wales, France), but information about father’s occupations were missing for some births in most countries, ranging from 3 to 26 %. The distribution of mothers’ occupations varied much more widely between countries – The percentage in the no occupation/student group ranged from 8 to 48 % and the percentage in the unskilled/skilled worker group varied from 1 to 45 %. The proportions of professionals/managers varied from 0.2 to 40 %. In general, the proportion of missing data was higher for occupation than educational level.

**Table 3:** Percentage distribution of mothers and fathers’ occupations and stillbirth rates by occupational group

Country	Total Births	No occupation or student	Skilled/ unskilled workers	Technicians/ clerical/ service	Managers/ professionals	Total Stillbirths (SB)	Overall SB rate	SB rate No occupation or student	SB rate Skilled/ unskilled workers	SB rate Technicians/ clerical/ service	SB rate Managers/ professionals
	N (all stated)	%	%	%	%	N	*p* 1000	*p* 1000	*p* 1000	*p* 1000	*p* 1000
Maternal occupation											
BE: Brussels	24432	46.6	7.4	42.2	3.8	127	5.2	6.1	5.0	4.5	2.1
BE: Flanders	64790	17.8	15.3	60.9	6.0	229	3.5	5.3	4.7	2.9	1.3
BE: Wallonia	35777	40.4	9.0	46.5	4.0	139	3.9	5.0	3.7	3.1	2.1
Estonia	11731	7.5	9.6	42.9	40.0	44	3.8	3.4	6.2	4.0	3.0
Finland	43734	11.8	12.4	53.7	22.1	128	2.9	2.9	2.2	3.1	2.8
France	14239	14.5	17.2	55.9	12.4	86	6.0	5.8	9.0	5.7	4.0
Germany	488041	34.0	45.1	17.0	3.9	1378	2.8	3.5	2.7	2.0	2.6
Ireland	70528	26.4	5.4	37.8	30.3	319	4.5	5.6	5.5	4.5	3.5
Lithuania	30568	29.0	8.8	26.5	35.6	137	4.5	5.6	3.7	3.5	4.5
Portugal	87627	19.9	19.4	43.1	17.7	211	2.4	3.9	3.4	1.6	1.6
Romania	199641	47.5	1.3	51.0	0.2	780	3.9	5.4	5.0	2.5	3.3
Slovenia	7687	0.0	5.0	58.4	36.6	18	2.3	0.0	2.6	1.6	3.6
Spain	465343	24.7	11.3	46.4	17.6	1305	2.8	3.7	3.4	2.4	2.4
Paternal occupation											
BE: Brussels	22976	20.9	22.4	43.1	13.6	106	4.6	7.9	3.7	4.3	1.9
BE: Flanders	62537	4.9	38.5	42.7	13.9	213	3.4	5.9	3.9	3.1	2.1
BE: Wallonia	30930	9.8	36.4	42.2	11.5	110	3.6	6.9	3.5	3.1	2.5
Estonia	12274	2.2	34.7	19.7	43.4	39	3.2	0.0	3.3	3.3	3.2
Finland	52555	3.7	40.7	30.7	24.8	88	1.7	0.0	1.8	2.0	1.3
France	13637	4.3	37.0	40.3	18.4	70	5.1	6.9	6.7	3.5	5.2
Ireland	54906	7.1	40.1	26.1	26.7	240	4.4	5.4	4.4	3.8	4.6
Portugal	90056	5.2	43.2	34.9	16.7	192	2.1	6.0	2.4	1.7	1.0
Spain	448911	1.5	46.8	32.0	19.6	1193	2.7	2.9	2.9	2.6	2.3
UK: England and Wales	636793	NA^a^	40.2	21.2	38.6	3053	4.8	NA*	5.5	4.7	4.1

^a^Students included with unclassified occupations, see Methods and note to Table 1

### Stillbirth rates and risk ratios by socioeconomic classification

**Fig. 1:**
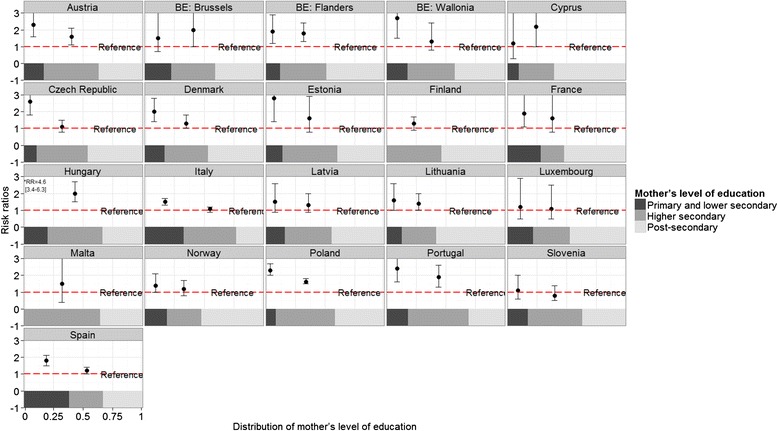
Stillbirth risk ratios according to mother’s level of education by country

**Fig. 2:**
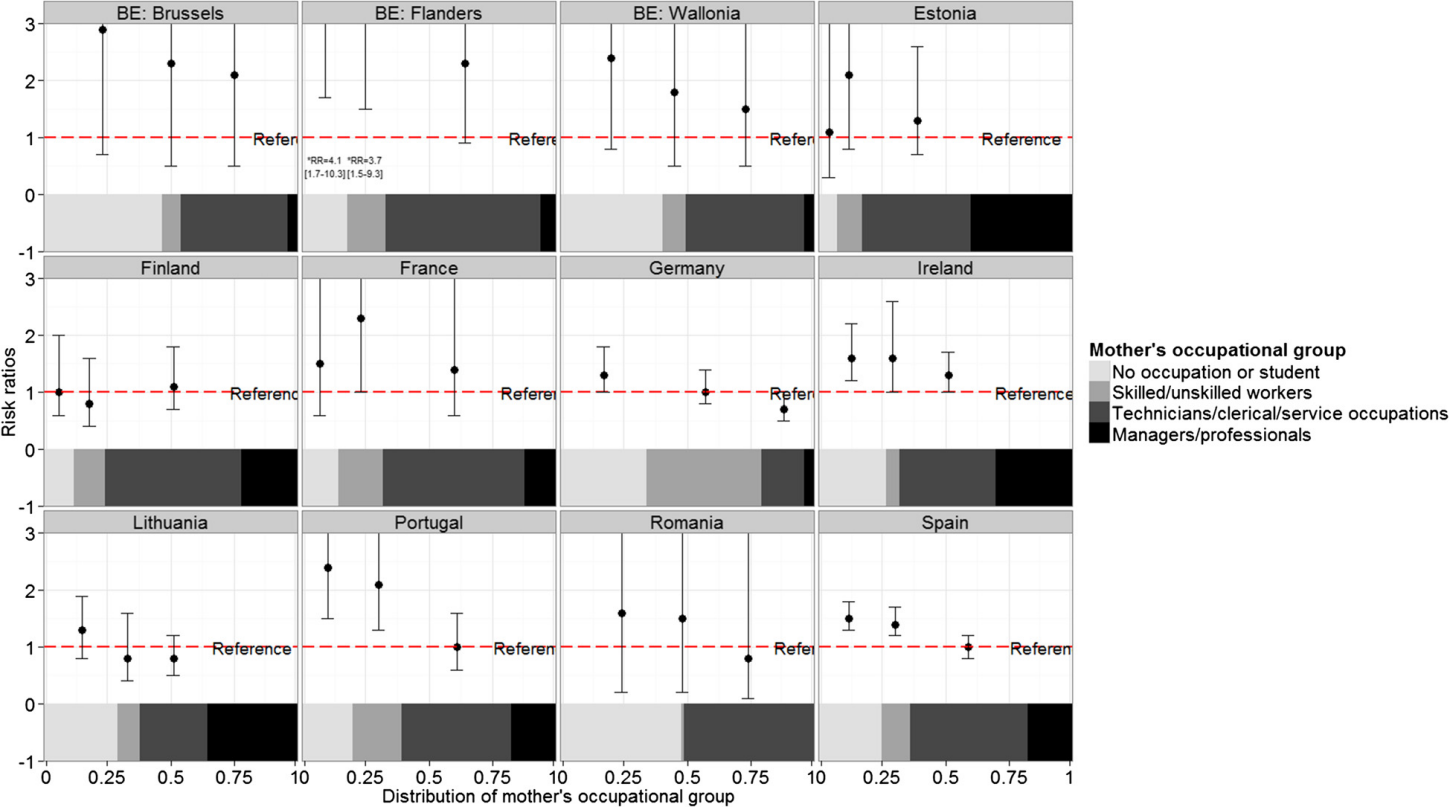
Stillbirth risk ratios according to mothers’ occupational group by country

**Fig. 3:**
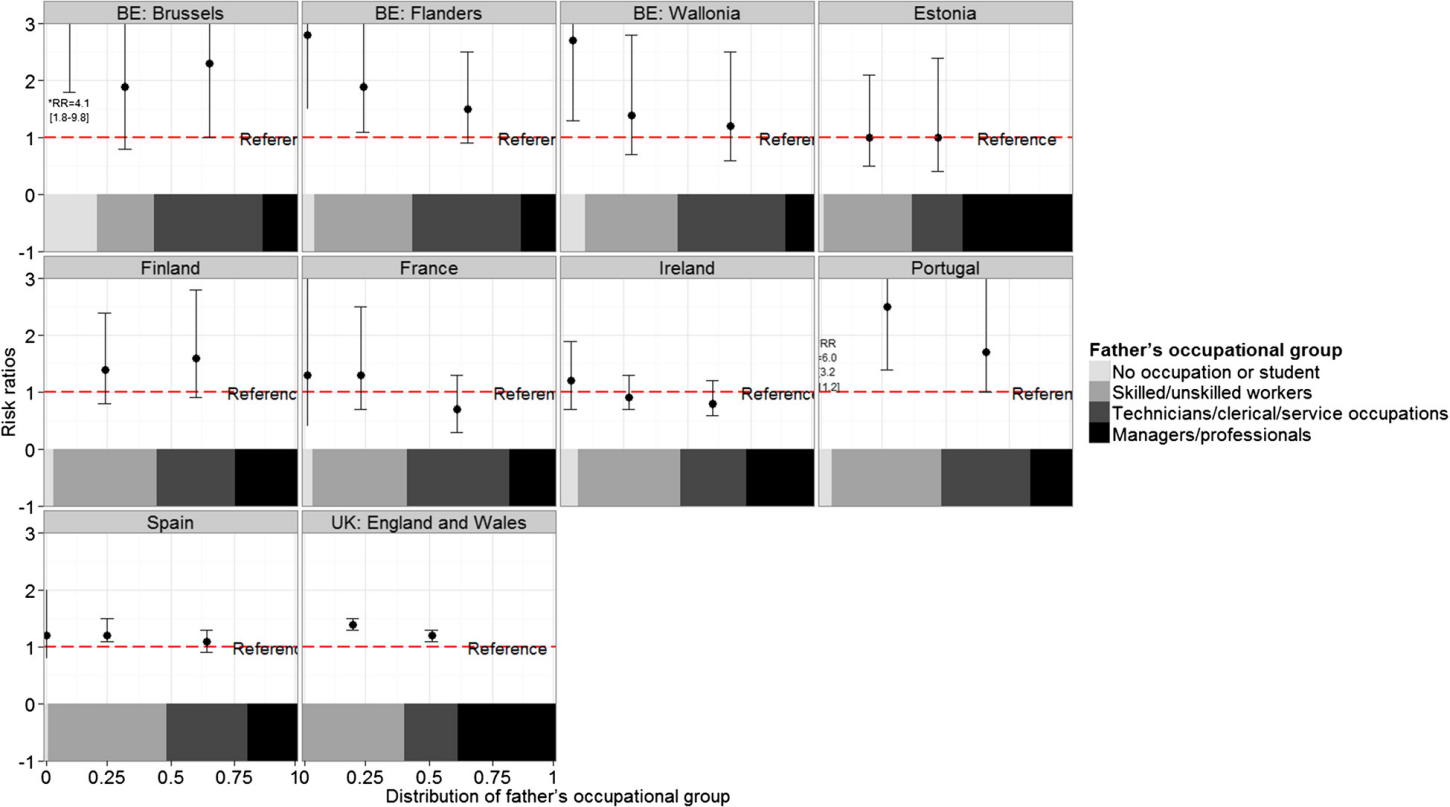
Stillbirth risk ratios according to father’s occupation by country

Stillbirth rates ranged from approximately 2 to 5 per 1000 in participating countries. Absolute rates for the socioeconomic classifications are displayed in Tables 2 and 3, while Figs. 1, 2 and 3 show RR of all categories compared to the highest category, juxtaposed with the proportional size of the groups illustrated by the band at the bottom of each country-specific graph. For mother’s level of education, the lowest rate was in the most highly educated group in all countries except for Slovenia. For the occupational classifications, relationships were less consistently monotonic and many RR were not significantly different from the reference category. However, countries mainly had the lowest stillbirth rate in the highest socioeconomic group, except Germany, Finland, Romania and Lithuania for mother’s occupational group and Estonia, France, and Ireland for father’s occupational group.

Compared to women with post-secondary education, women with primary and lower secondary education had a median RR of stillbirth of 1.9 (IQR: 1.5 to 2.4) in the 19 countries providing these data. For women with higher secondary education, the median RR was 1.4 (IQR: 1.2 to 1.6). For mother’s occupational group, the median RR comparing outcomes among manual workers with managers and professionals was 1.6 (IQR: 1.0–2.1) whereas for father’s occupational group, the median RR was 1.4 (IQR: 1.2–1.8). Estimates of RR and 95 % confidence intervals are given in Additional file 1: Tables S2, S3 and S4. The magnitude of the RR for the three socioeconomic variables was not correlated with the percent of missing data (rho statistics and *p* values presented in Additional file 1).

**Fig. 4:**
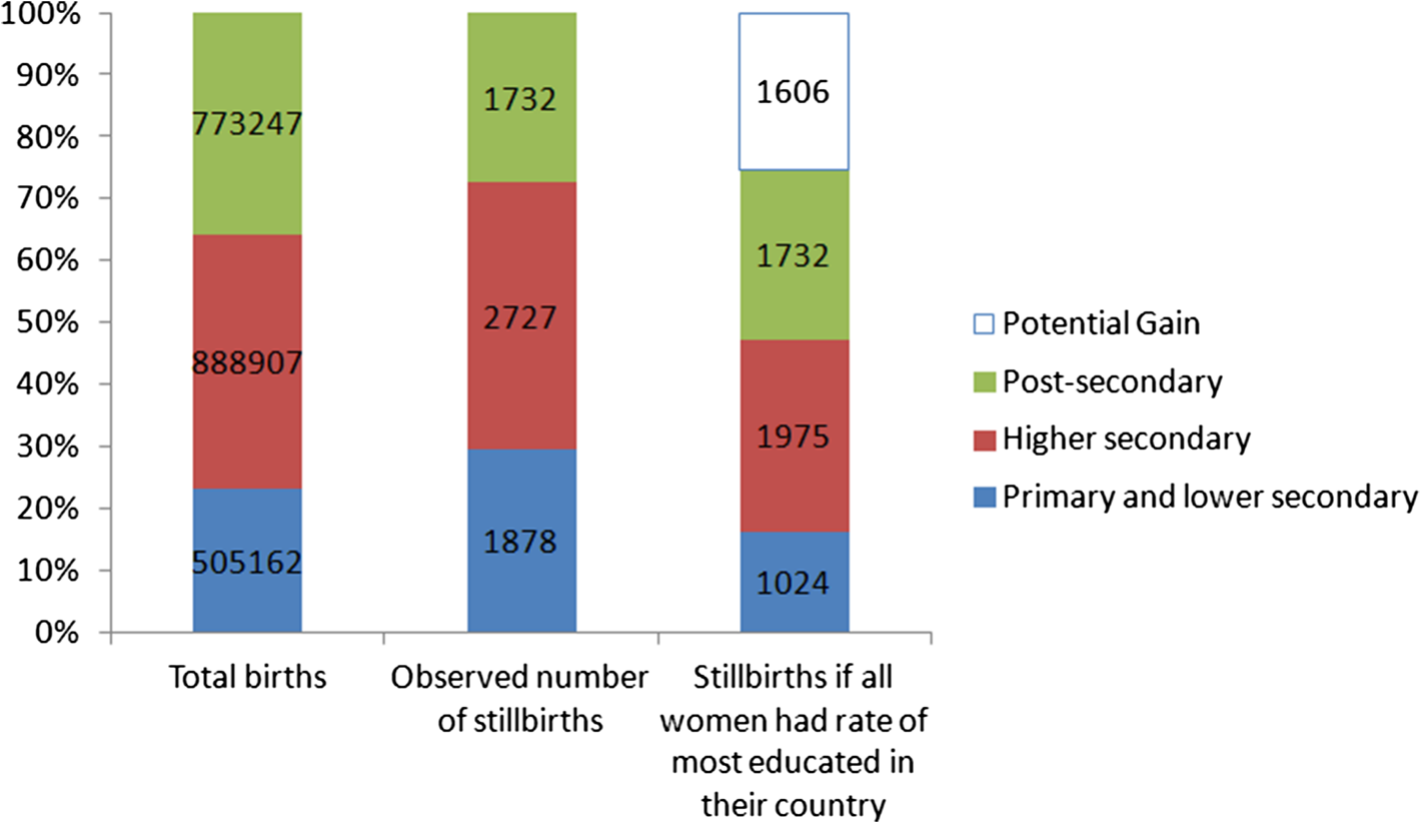
Total births, observed stillbirths and predicted stillbirths if all women experienced the stillbirth rates of women with post-secondary schooling in 19 European countries in 2010

Table 4 presents %PAR for each socioeconomic classification by country. This measure assesses the difference between the observed situation and an optimal one in which all pregnant women have the stillbirth risks of the group with the highest educational or occupational level. For mother’s level of education, the median %PAR was 26 (IQR 16 to 31). For Father’s and mother’s occupational groups, medians (IQR) were 19 (3 to 37) and 22 (12 to 37) respectively. When applied to the entire sample with data on mother’s level of education, 1606 out of 6337 stillbirths would not have occurred if stillbirth rates for all women were the same as for women with post-secondary education in their country (Fig. 4). For father’s occupational groups, 904 of 5304 stillbirths would not have occurred if all groups had the same stillbirth rates as professionals and managers while using mother’s occupational groups in the same way, 861 out of 4901 would not have occurred.

**Table 4:** Population attributable risk (PAR) percentage by country

Country	Education		Maternal occupation		Paternal occupation	
	% PAR	95 % CI	% PAR	95 % CI	% PAR	95 % CI
Austria	32	15–46				
BE: Brussels	33	−9–58	59	−63–90	59	10–81
BE: Flanders	29	13–42	64	14–85	39	5–61
BE: Wallonia	31	1–51	46	−65–82	29	−33–62
Cyprus	27	−13–53				
Czech Republic	17	1–31				
Denmark	22	7–35				
Estonia	34	−1–56	20	−23–48	0	−44–30
Finland	11	−5–24	5	−33–32	22	−19–49
France	27	0–47	34	−34–68	−1	−65–38
Germany			7	−22–29		
Hungary	54	41–64				
Ireland			24	7–37	−6	−30–13
Italy	15	6–24				
Latvia	17	−6–36				
Lithuania	16	1–30	0	−26–20		
Luxembourg	5	−42–37				
Malta	24	−100–71				
Norway	12	−4–25				
Poland	30	25–35				
Portugal	41	22–55	33	3–54	53	24–71
Romania			15	−499–88		
Slovenia^a^	−7	−42–20				
Spain	26	17–33	14	2–25	13	2–24
UK: England and Wales					15	11–19

^a^Terminations of pregnancy removed for Slovenia

## Discussion

Despite the differences in health policies, educational policies and reporting systems across Europe, the available data from routine systems about stillbirths by socioeconomic status pointed to widespread and consistent socioeconomic inequalities in the stillbirth rate. Over three quarters of 29 participating European countries provided data on stillbirths by either mothers’ educational levels or parents’ occupation. While data about mothers’ educational levels were more widely available and comparable than data about mothers’ or fathers’ occupations, some of the larger countries, notably Germany and England and Wales combined only had data by parents’ occupations. Among countries with data, the median RR for stillbirth was 1.9 for women with primary or lower secondary education and 1.4 for those the intermediate category of higher secondary education compared to women with post-secondary education.. Median RR in the lowest occupational categories compared to highest were 1.6 and 1.4, respectively, for mothers’ and fathers’ occupational groups. These differences had substantial impacts at a population-level: if all women faced the stillbirth risks of the most educated, the number of stillbirths would be 25 % lower.

The main strength of our study is the ability to present data from all over Europe collected for the same year using a standardised instrument. We also requested data about mothers’ educational levels using local classifications and mapped these to the ISCED-97 classification to ensure consistency in coding and then checked our coded data with the data providers in each country. Our study also has several limitations. Because we used aggregated data, we were unable to explore the contribution of other demographic or behavioural factors, such as maternal age, parity, migrant status, smoking or body mass index to the higher risks associated with our socioeconomic indicators. Further, even though Euro-Peristat uses a common inclusion threshold of 22 weeks of gestation for births and deaths, not all countries are able to provide these data and even when they can, practices related to recording of early stillbirths differ [19]. While it is likely that these differences relate primarily to legislation and recording practices and not women’s socioeconomic circumstances, social inequalities may be more acute for early stillbirths and underreporting might thus affect the comparability between countries [5]. For countries where TOP cannot be differentiated from spontaneous stillbirths, the social gradient may be attenuated as TOP reflect the prevalence of anomalies for which the social gradient is less consistent [23, 24] as well as access to prenatal screening, which may have an inverse gradient in some countries [25]. Finally, some countries had missing data on socioeconomic factors which could bias the estimates. This bias likely leads to underestimates of the effects as missing data were more frequent among stillbirths and may also be more frequent for socially disadvantaged and migrant women.[26]

Because there are no universal measures of social disadvantage, researchers use a wide variety of different indicators: occupation, education, income, other measures of wealth, housing conditions, lack of access to health care, in particular prenatal care, and others. We used two indicators selected in a Delphi process based on assessments of importance and feasibility. The definition of these indicators, level of education and occupational group, were based on common classifications agreed upon by international organizations. However, categories may not reflect similar constructs in all countries or be understood in the same manner by responders or coders.

For mothers’ educational level, comparability issues related to the limit for differentiating between lower and higher secondary schooling, to the classification of vocational tracts and to the inclusion of a category for no or limited schooling, which may be relevant for some migrant women There are also real differences in educational attainment across Europe. Similarly broad variations in maternal education have been documented in comparative studies based on birth cohorts across Europe [27, 28]. However, despite the differences in the distribution of maternal educational attainment a negative social gradient was observed in most countries when this indicator was used.

For mothers’ and father’ occupation, questions of comparability were more complex as it was not possible to describe original data. Rules for recording and classifying occupations for parents who are not in paid employment or seeking work at the time their babies are born and lone mothers registering births without the involvement of the babies’ fathers were not clear. Compared to mothers’ educational level, there was higher variation in the distribution of categories between countries, RR tended to be lower and did not always follow a linear gradient. Furthermore, while the ISCO classification appeared to be widely used, there are questions about whether a status based classification of occupations is most relevant. Some research suggests that a classification based on employment relations (i.e. employers, employees or self-employed) provides more useful insights into the nature of social inequalities and their potential impact of health [29, 30] and these principles have been used for the development of a European Classification [31]. Given these conceptual and practical difficulties, one way of improving comparisons across countries would be to promote the recording of mothers’ educational level in routine systems either directly or through data linkage.

Studies from other high-income countries have documented elevated risks of similar magnitude for women with low socioeconomic position. For instance, using Canadian data, Auger et al. reported relative risks of 1.6 and 1.3 for low and intermediate educational levels compared with women with the highest educational level [5]. In a large US case control study, the unadjusted OR for educational level were 1.5 and 1.4, respectively for women with primary or some secondary and completed secondary compared to women with a post-secondary education [32]. The reasons for these elevated risks are likely multiple and interconnected. One UK study investigating stillbirths by area-based deprivation scores found that deprivation gaps existed for all causes, except for mechanical events, with the widest gap for stillbirths due to antepartum haemorrhages [3]. The study in Canada found differences throughout the gestational age spectrum, although they were more marked at earlier gestational ages [5].

The finding that social factors are not restricted to specific causes or gestational age groups is not surprising. Many risk factors, including smoking, diet and healthcare factors, affect a number of pregnancy complications such as extremely preterm delivery, growth restriction and congenital anomalies which raise stillbirth risk and are more common among less advantaged groups in the population. Mothers in these groups are more likely to have high body mass indices (BMI) and to smoke during pregnancy and these are important risk factors for stillbirth [33]. Other risk factors, such as migrant status, also affect a wide range of reproductive health outcomes [34, 35]. Social differences in screening and termination of pregnancy may also play a role if socially disadvantaged women are less likely to terminate pregnancies with lethal anomalies, either because of lack of access to screening or differences in attitudes to pregnancy terminations [24, 25, 36]. Antenatal detection of growth restriction may play a role in preventing stillbirth and the extent to which social position affects access to screening should also be considered [37]. The question of how much of the social gradient is explained by these behavioural and healthcare factors, especially those that are potentially modifiable, is an important area for further research.

While we found consistent associations between risks of stillbirth and social factors in European countries, the magnitude of the social gradient varied. Some of this variation could be related to small sample sizes in some countries as well as to the non-comparability of socioeconomic classifications. However, studies comparing the Nordic countries have found differences in the social gradient of stillbirth risk even within these relatively homogenous societies [2]. Other studies of socioeconomic inequalities in Europe have uncovered differences in the inequality gradient for overall mortality rates which are partially correlated with differences in the prevalence of smoking and overweight in the population [38]. Other European studies have also suggested that healthcare can contribute to differences in outcomes, for instance in cancer mortality, which may reflect social inequalities in access and quality of care [39]. In the perinatal field, this is an important area for further investigation and could be a powerful tool for identifying population-based risk factors and health care policies that contribute to stillbirth etiology and thereby to prevention of stillbirth for the benefit of all women.

## Conclusion

This study documented widespread social inequalities in stillbirth risk in Europe, revealing the extent to which stillbirth can be a consequence of social disadvantage. While more research is needed to better understand differences in the magnitude of these differences between countries and how to harmonise use of socioeconomic categories, available data in routine systems can be used to set goals for the future and to monitor changes over time.

## Availability of data and materials

Data used in this analysis can be requested from the Euro-Peristat coordination team through the project’s website (www.europeristat.com).

## Additional file

Additional file 1:
**Table S1.** Inclusion criteria for stillbirths. **Table S2.** Stillbirth risk ratios according to maternal education by country. **Table S3.** Stillbirth risk ratios according to maternal occupation by country. **Table S4.** Stillbirth risk ratios according to paternal occupation by country. (DOCX 37 kb)
