# Supplementary material for: Socioeconomic inequalities in stillbirth rates in Europe: measuring the gap using routine data from the Euro-Peristat Project
Source: BMC Pregnancy Childbirth. 2016 Jan 19;16:15. doi: 10.1186/s12884-016-0804-4 (PMC4727282; doi:10.1186/s12884-016-0804-4)
Supplement: Additional file 1: — Table S1. Inclusion criteria for stillbirths. Table S2. Stillbirth risk ratios according to maternal education by country. Table S3. Stillbirth risk ratios according to maternal occupation by country. Table S4. Stillbirth risk ratios according to paternal occupation by country. (DOCX 37 kb) [file 12884_2016_804_MOESM1_ESM.docx]

Suppementary table (online appendix) Table S1 – Inclusion criteria for stillbirths

NOTE: lower inclusion criteria may be used in individual countries, but Euro-Peristat requests data starting at 22 weeks from all countries. Euro-Peristat also request data for stillbirths and terminations of pregnancy separately, but some countries, as noted, could not distinguish between spontaneous stillbirths and TOP.

| Country/coverage | inclusion criteria for fetal deaths  As requested for Euro-Peristat, if not criteria |
| --- | --- |
| Belgium |  |
| BE: Brussels | Yes TOPS included |
| BE: Flanders | Yes – TOP included |
| BE: Wallonia | Yes– TOP included |
| Czech Republic | Yes– TOP included |
| Denmark | Yes |
| Germany | 500+ grams |
| Estonia | Yes |
| Ireland | 500+ grams or 24+ weeks |
| Greece (2009) | 24+ weeks |
| Spain | 180 days |
| France | Yes |
| FR: regional register | Yes |
| Italy | Yes |
| Cyprus (2007) | 22+ weeks perinatal register; 28+ weeks death register – TOP included |
| Latvia | Yes |
| Lithuania | Yes |
| Luxembourg | Yes – TOP included |
| Hungary | legal 24 wks or 500g; fetal deaths and TOP at 22-23wks included |
| Malta | Yes |
| Netherlands | Yes – TOPs included |
| Austria | 500+ grams |
| Poland | 500+ grams |
| Portugal | 24+ weeks, voluntary data at 22-23 weeks |
| Romania | Yes |
| Slovenia | 500+ grams |
| Slovakia | Yes |
| Finland | Yes |
| Sweden | Yes |
| United Kingdom |  |
| UK: England and Wales | 24+ weeks, TOP should be included |
| UK: Scotland | 24* weeks; incomplete voluntary notification at 22-23 weeks, TOP should be included |
| UK: Northern Ireland | 24+ weeks |
| Iceland | Yes – TOP included |
| Norway | Yes |
| Switzerland | Yes |

Supplementary table 2 (online appendix): Stillbirth risk ratios according to maternal education by country

| **Country** | **Maternal education** | **Relative Risks** | **95% CI** |
| --- | --- | --- | --- |
| Austria | Primary and lower secondary | 2.3 | 1.6-3.3 |
| Austria | Higher secondary | 1.6 | 1.1-2.1 |
| Austria | Post-secondary | Reference |  |
| BE: Brussels | Primary and lower secondary | 1.5 | 0.7-3.5 |
| BE: Brussels | Higher secondary | 2.0 | 1.0-4.0 |
| BE: Brussels | Post-secondary | Reference |  |
| BE: Flanders | Primary and lower secondary | 1.9 | 1.2-2.9 |
| BE: Flanders | Higher secondary | 1.8 | 1.3-2.4 |
| BE: Flanders | Post-secondary | Reference |  |
| BE: Wallonia | Primary and lower secondary | 2.7 | 1.5-4.8 |
| BE: Wallonia | Higher secondary | 1.3 | 0.8-2.4 |
| BE: Wallonia | Post-secondary | Reference |  |
| Cyprus | Primary and lower secondary | 1.2 | 0.3-5.2 |
| Cyprus | Higher secondary | 2.2 | 1.0-5.0 |
| Cyprus | Post-secondary | Reference |  |
| Czech Republic | Primary and lower secondary | 2.6 | 1.8-3.8 |
| Czech Republic | Higher secondary | 1.1 | 0.8-1.5 |
| Czech Republic | Post-secondary | Reference |  |
| Denmark | Primary and lower secondary | 2.0 | 1.4-2.8 |
| Denmark | Higher secondary | 1.3 | 1.0-1.8 |
| Denmark | Post-secondary | Reference |  |
| Estonia | Primary and lower secondary | 2.8 | 1.4-5.6 |
| Estonia | Higher secondary | 1.6 | 0.8-2.9 |
| Estonia | Post-secondary | Reference |  |
| Finland | Primary and lower secondary | -- | -- |
| Finland | Higher secondary | 1.3 | 0.9-1.7 |
| Finland | Post-secondary | Reference |  |
| France | Primary and lower secondary | 1.9 | 1.1-3.4 |
| France | Higher secondary | 1.6 | 0.8-3.1 |
| France | Post-secondary | Reference |  |
| Hungary | Primary and lower secondary | 4.6 | 3.4-6.3 |
| Hungary | Higher secondary | 2.0 | 1.5-2.7 |
| Hungary | Post-secondary | Reference |  |
| Italy | Primary and lower secondary | 1.5 | 1.3-1.7 |
| Italy | Higher secondary | 1.1 | 0.9-1.2 |
| Italy | Post-secondary | Reference |  |
| Latvia | Primary and lower secondary | 1.5 | 0.9-2.6 |
| Latvia | Higher secondary | 1.3 | 0.9-2.0 |
| Latvia | Post-secondary | Reference |  |
| Lithuania | Primary and lower secondary | 1.6 | 1.0-2.6 |
| Lithuania | Higher secondary | 1.4 | 1.0-2.0 |
| Lithuania | Post-secondary | Reference |  |
| Luxembourg | Primary and lower secondary | 1.2 | 0.5-2.9 |
| Luxembourg | Higher secondary | 1.1 | 0.5-2.5 |
| Luxembourg | Post-secondary | Reference |  |
| Malta | Primary and lower secondary | -- | -- |
| Malta | Higher secondary | 1.5 | 0.4-5.6 |
| Malta | Post-secondary | Reference |  |
| Norway | Primary and lower secondary | 1.4 | 1.0-2.1 |
| Norway | Higher secondary | 1.2 | 0.8-1.7 |
| Norway | Post-secondary | Reference |  |
| Poland | Primary and lower secondary | 2.3 | 2.0-2.7 |
| Poland | Higher secondary | 1.6 | 1.5-1.8 |
| Poland | Post-secondary | Reference |  |
| Portugal | Primary and lower secondary | 2.4 | 1.6-3.6 |
| Portugal | Higher secondary | 1.9 | 1.3-2.6 |
| Portugal | Post-secondary | Reference |  |
| Slovenia* | Primary and lower secondary | 1.1 | 0.6-2.0 |
| Slovenia* | Higher secondary | 0.8 | 0.5-1.4 |
| Slovenia* | Post-secondary | Reference |  |
| Spain | Primary and lower secondary | 1.8 | 1.5-2.1 |
| Spain | Higher secondary | 1.2 | 1.0-1.4 |
| Spain | Post-secondary | Reference |  |

* Terminations of pregnancy removed for Slovenia

NOTE: The Spearman rho for the RR of the lowest social category to the reference group with the proportion of missing data was: 0.02 (p=0.93).

Supplementary table 3 (online appendix): Stillbirth risk ratios according to maternal occupation by country

| **Country** | **Maternal occupation** | **Relative Risks** | **95% CI** |
| --- | --- | --- | --- |
| BE: Brussels | No occupation or student | 2.9 | 0.7-11.7 |
| BE: Brussels | Skilled/unskilled workers | 2.3 | 0.5-10.7 |
| BE: Brussels | Technicians/clerical/service | 2.1 | 0.5-8.6 |
| BE: Brussels | Managers/professionals | Reference |  |
| BE: Flanders | No occupation or student | 4.1 | 1.7-10.3 |
| BE: Flanders | Skilled/unskilled workers | 3.7 | 1.5-9.3 |
| BE: Flanders | Technicians/clerical/service | 2.3 | 0.9-5.6 |
| BE: Flanders | Managers/professionals | Reference |  |
| BE: Wallonia | No occupation or student | 2.4 | 0.8-7.6 |
| BE: Wallonia | Skilled/unskilled workers | 1.8 | 0.5-6.3 |
| BE: Wallonia | Technicians/clerical/service | 1.5 | 0.5-4.7 |
| BE: Wallonia | Managers/professionals | Reference |  |
| Estonia | No occupation or student | 1.1 | 0.3-4.0 |
| Estonia | Skilled/unskilled workers | 2.1 | 0.8-5.1 |
| Estonia | Technicians/clerical/service | 1.3 | 0.7-2.6 |
| Estonia | Managers/professionals | Reference |  |
| Finland | No occupation or student | 1.0 | 0.6-2.0 |
| Finland | Skilled/unskilled workers | 0.8 | 0.4-1.6 |
| Finland | Technicians/clerical/service | 1.1 | 0.7-1.8 |
| Finland | Managers/professionals | Reference |  |
| France | No occupation or student | 1.5 | 0.6-3.7 |
| France | Skilled/unskilled workers | 2.3 | 1.0-5.3 |
| France | Technicians/clerical/service | 1.4 | 0.6-3.2 |
| France | Managers/professionals | Reference |  |
| Germany | No occupation or student | 1.3 | 1.0-1.8 |
| Germany | Skilled/unskilled workers | 1.0 | 0.8-1.4 |
| Germany | Technicians/clerical/service | 0.7 | 0.5-1.0 |
| Germany | Managers/professionals | Reference |  |
| Ireland | No occupation or student | 1.6 | 1.2-2.2 |
| Ireland | Skilled/unskilled workers | 1.6 | 1.0-2.6 |
| Ireland | Technicians/clerical/service | 1.3 | 1.0-1.7 |
| Ireland | Managers/professionals | Reference |  |
| Lithuania | No occupation or student | 1.3 | 0.8-1.9 |
| Lithuania | Skilled/unskilled workers | 0.8 | 0.4-1.6 |
| Lithuania | Technicians/clerical/service | 0.8 | 0.5-1.2 |
| Lithuania | Managers/professionals | Reference |  |
| Portugal | No occupation or student | 2.4 | 1.5-3.8 |
| Portugal | Skilled/unskilled workers | 2.1 | 1.3-3.4 |
| Portugal | Technicians/clerical/service | 1.0 | 0.6-1.6 |
| Portugal | Managers/professionals | Reference |  |
| Romania | No occupation or student | 1.6 | 0.2-11.5 |
| Romania | Skilled/unskilled workers | 1.5 | 0.2-11.5 |
| Romania | Technicians/clerical/service | 0.8 | 0.1-5.4 |
| Romania | Managers/professionals | Reference |  |
| Spain | No occupation or student | 1.5 | 1.3-1.8 |
| Spain | Skilled/unskilled workers | 1.4 | 1.2-1.7 |
| Spain | Technicians/clerical/service | 1.0 | 0.8-1.2 |
| Spain | Managers/professionals | Reference |  |

NOTE: The Spearman rho for the RR of the lowest social category to the reference group with the proportion of missing data was: -0.45 (p=0.14).

Supplementary table 4: Stillbirth risk ratios according to paternal occupation by country

| **Country** | **Paternal occupation** | **Relative Risks** | **95% CI** |
| --- | --- | --- | --- |
| BE: Brussels | No occupation or student | 4.1 | 1.8-9.8 |
| BE: Brussels | Skilled/unskilled workers | 1.9 | 0.8-4.8 |
| BE: Brussels | Technicians/clerical/service | 2.3 | 1.0-5.3 |
| BE: Brussels | Managers/professionals | Reference |  |
| BE: Flanders | No occupation or student | 2.8 | 1.5-5.5 |
| BE: Flanders | Skilled/unskilled workers | 1.9 | 1.1-3.1 |
| BE: Flanders | Technicians/clerical/service | 1.5 | 0.9-2.5 |
| BE: Flanders | Managers/professionals | Reference |  |
| BE: Wallonia | No occupation or student | 2.7 | 1.3-5.9 |
| BE: Wallonia | Skilled/unskilled workers | 1.4 | 0.7-2.8 |
| BE: Wallonia | Technicians/clerical/service | 1.2 | 0.6-2.5 |
| BE: Wallonia | Managers/professionals | Reference |  |
| Estonia | No occupation or student | -- | -- |
| Estonia | Skilled/unskilled workers | 1.0 | 0.5-2.1 |
| Estonia | Technicians/clerical/service | 1.0 | 0.4-2.4 |
| Estonia | Managers/professionals | Reference |  |
| Finland | No occupation or student | -- | -- |
| Finland | Skilled/unskilled workers | 1.4 | 0.8-2.4 |
| Finland | Technicians/clerical/service | 1.6 | 0.9-2.8 |
| Finland | Managers/professionals | Reference |  |
| France | No occupation or student | 1.3 | 0.4-4.1 |
| France | Skilled/unskilled workers | 1.3 | 0.7-2.5 |
| France | Technicians/clerical/service | 0.7 | 0.3-1.3 |
| France | Managers/professionals | Reference |  |
| Ireland | No occupation or student | 1.2 | 0.7-1.9 |
| Ireland | Skilled/unskilled workers | 0.9 | 0.7-1.3 |
| Ireland | Technicians/clerical/service | 0.8 | 0.6-1.2 |
| Ireland | Managers/professionals | Reference |  |
| Portugal | No occupation or student | 6.0 | 3.2-11.2 |
| Portugal | Skilled/unskilled workers | 2.5 | 1.4-4.2 |
| Portugal | Technicians/clerical/service | 1.7 | 1.0-3.1 |
| Portugal | Managers/professionals | Reference |  |
| Spain | No occupation or student | 1.2 | 0.8-2.0 |
| Spain | Skilled/unskilled workers | 1.2 | 1.1-1.5 |
| Spain | Technicians/clerical/service | 1.1 | 0.9-1.3 |
| Spain | Managers/professionals | Reference |  |
| UK: England and Wales | No occupation or student | -- | -- |
| UK: England and Wales | Skilled/unskilled workers | 1.4 | 1.3-1.5 |
| UK: England and Wales | Technicians/clerical/service | 1.2 | 1.1-1.3 |
| UK: England and Wales | Managers/professionals | Reference |  |

NOTE: The Spearman rho for the RR of the lowest social category to the reference group with the proportion of missing data was: -0.19 (p=0.68).
